# Comparative proteomics analysis of *Shiraia bambusicola* revealed a variety of regulatory systems on conidiospore formation

**DOI:** 10.3389/fmicb.2024.1373597

**Published:** 2024-05-22

**Authors:** Wen Du, Chunlong Sun, Tao Wu, Wang Li, Bin Dong, Baogui Wang, Shuai Shang, Qian Yang, Wenwen Huang, Shaopeng Chen

**Affiliations:** ^1^School of Biological and Environmental Engineering, Shandong University of Aeronautics, Binzhou, China; ^2^Binzhou Key Laboratory of Chemical Drug R&D and Quality Control, Binzhou, China

**Keywords:** *Shiraia bambusicola*, conidia, differential proteome, carbon metabolism, glycoside hydrolase

## Abstract

*Shiraia bambusicola* is a typical parasitic medicinal fungus of the family Shiraiaceae. The fruiting bodies of *S. bambusicola* cannot be cultivated artificially, and active substances can be effectively produced via fermentation. The mechanism of conidia production is a research hotspot in the industrial utilization and growth development of *S. bambusicola*. This study is the first to systematically study the proteomics of conidiospore formation from *S. bambusicola.* Near-spherical conidia were observed and identified by internal transcribed spacer (ITS) sequence detection. A total of 2,840 proteins were identified and 1,976 proteins were quantified in the mycelia and conidia of *S. bambusicola*. Compared with mycelia, 445 proteins were differentially expressed in the conidia of *S. bambusicola*, with 165 proteins being upregulated and 280 proteins being downregulated. The Gene Ontology (GO) annotation results of differential proteomics showed that the biological process of *S. bambusicola* sporulation is complex. The Kyoto Encyclopedia of Genes and Genomes (KEGG) metabolic pathway analysis showed that the differential proteins were mainly involved in starch and sucrose metabolism, biosynthesis of secondary metabolites, microbial metabolism in diverse environments, and other processes. Our in-depth speculative analysis showed that proteins related to carbohydrate metabolism were differentially expressed in conidiospore formation of *S. bambusicola*, suggesting the involvement of saccharides. Conidiation may increase the synthesis and release of ethanol and polysaccharide proteins such as glycoside hydrolase (GH), suppress host immunity, and facilitate *S. bambusicola* to infect and colonize of the host. In-depth analysis of differential proteomes will help reveal the molecular mechanism underlying the conidiospore formation of *S. bambusicola*, which has strong theoretical and practical significance.

## Introduction

*Shiraia bambusicola* Henn. is a parasitic fungus on young bamboo branches of *Brachystachyum* (Dai et al., 2019). *Shiraia* is a monotypic genus. *Shiraia bambusicola* is the type species of this genus and belongs to the Shiraiaceae family in Pleosporales (Shen et al., 2015; Tao et al., 2021). *Shiraia bambusicola* is an important traditional Chinese medicine resource. *Shiraia bambusicola* wine is commonly used to treat cold stomachache, rheumatoid arthritis, tracheitis, bruises, anemia, and headaches (Ren et al., 2022). Decoction in water has a certain therapeutic effect on the recovery period of acute hepatitis and chronic hepatitis (Xia et al., 2022). In recent years, the demand for *S. bambusicola* in the market has greatly increased with the recognition of its medicinal value. The current research and utilization of *S. bambusicola* mainly relies on collection in the wild. Yield and quality are strongly affected by factors such as climate, distribution, and harvesting season (Bu and Yang, 2020). The artificial collection of natural resources is rapidly increasing, and there is a serious lack of basic biological knowledge and protection awareness of *S. bambusicola*, resulting in the decline of this species. Exploring the industrial production of *S. bambusicola* is an ideal way to solve this problem.

Fungi can reproduce asexually through fragmentation, budding, and sporulation, and they can reproduce sexually through the fusion and meiosis of gametes to produce sexual spores (Hu et al., 2023). Some fungi reproduce only asexually, but most fungi have a sexual life cycle (Bao, 2020). *Shiraia bambusicola* growing naturally on the host bamboo has a sexual life history, and it also undergoes an asexual life cycle. Nevertheless, *S. bambusicola* exclusively reproduces asexually in artificial culture (Han et al., 2012). The perithecium containing ascospores and the pycnidia producing phialoconidia were observed in the fruiting body of wild *S. bambusicola*. Besides the similar morphology of the phialoconidia and ascospores, elliptical to nearly spherical, transparent conidia were also observed (Han et al., 2010). *Shiraia bambusicola* cannot form fruiting bodies in artificial mediums, but only forms a natural *S. bambusicola*-like culture and nearly spherical conidia (Han et al., 2012; Tao et al., 2021). *Cordyceps sinensis* cannot cultivate fruiting bodies on artificial medium. Its breeding technology mainly induces the production of conidia through solid fermentation of *C. sinensis* (Bhetwal et al., 2021). Only by infecting *Hepialus armoricanus* larvae with conidia can the sexual reproduction process of *C. sinensis* be carried out (Xie et al., 2021). The loss of ergot alkaloid’s formation ability in *Claviceps purpurea* is often related to the shape of the conidia (Uhlig et al., 2021). The artificial culture of *S. bambusicola* has no pycnidia and phialospores, conidial structure is relatively simple. In the culture of fungi with relatively similar morphology, cytoplasmic proteins and acyl-coenzyme A binding protein regulated conidiation and pathogenicity in *Magnaporthe oryzae* (Cao et al., 2023). The lysine crotonylation proteins were significantly involved in various metabolic and biosynthetic processes of *Trichophyton rubrum* conidial stages (Xu et al., 2022). Therefore, the study on the morphological characteristics and individual development of *S. bambusicola* is helpful for the identification, infection cycle, and artificial inoculation and culture.

With the development and application of *S. bambusicola* and its active ingredients, new discoveries have been made in many research aspects. Most scholars focused on the production of hypocrellins, anthraquinone compounds, laccase, and polysaccharides, isolated the active substances, optimized the composition of the medium and culture conditions (Zhao et al., 2021). In addition, red light and associated bacteria have been found to promote the synthesis of secondary metabolites of *S. bambusicola*, and the yield has been reported to increase by several times to dozens of times (Ma et al., 2019; Wang et al., 2022). Although the artificial culture of *S. bambusicola* has become a research hotspot and has made certain progress, many problems remain to be addressed. For example, ① the mechanism of the synthetic hypocrellins is unknown, the biosynthetic pathway gene cloning, and gene expression of hypocrellins in *S. bambusicola* have not been fully elucidated. ② The content of active metabolites is low, and large-scale industrial production remains a distant goal (Wen et al., 2022). Conidiation is the main approach of asexual reproduction of *S. bambusicola*. In the biosynthetic industry, conidia serve as the first step in seed culture, which is an important preparation for the large-scale production of industrial fungi. The formation of fungal secondary metabolites is often related to its biological characteristics. The conidia produced by *Aspergillus niger* are accompanied with an increase in the amount of mycelial protein and glucoamylase activity (Driouch et al., 2010). The conidial formation ability of industrial fungi is of great significance for reducing production costs (Yang et al., 2022a), exploring its relationship with the production of active substances, controlling pollution, and preserving strains. Exploring methods to increase conidial production and its regulatory mechanisms is one of the effective ways to solve the problems.

Although studies on conidiospore formation in *S. bambusicola* are warranted, the proteome related to the conidial production of *S. bambusicola* has not been reported. To explore the protein molecular mechanism of conidiation from *S. bambusicola*, we observed and detected the characteristics of conidia of *S. bambusicola* and used enzymatic mass spectrometry (MS) to systematically analyze the proteome differences of conidiospore formation for the first time. The research will help determine the metabolism and molecules preferentially used by mycelia and conidial cells, understand new information on conidial formation, and provide a solid theoretical basis for subsequent in-depth research on *S. bambusicola*.

## Materials and methods

### Microorganism, media, and chemicals

*Shiraia bambusicola* strain BZ16 was isolated and identified by the Biopharmaceutical Teaching and Research Office in Shandong University of Aeronautics. This strain was deposited in the China Typical Culture Collection Center under strain deposit number: CCTCC NO: M209141. We cultivated *S. bambusicola* in potato dextrose agar (PDA) for 7 days and transferred it to a 500 mL Erlenmeyer flask containing 150 mL of Czapek medium. The flask was cultured at 26°C and 120 r/min for 10 days. The mycelia were collected and stored at −80°C. Czapek medium and PDA medium were prepared according to conventional methods. Relevant reagents for protein and DNA extraction were purchased from Tiangen Biotechnology Company. All other chemicals and reagents were of analytical grade.

### Conidia harvesting

On the 14th day after inoculation, the mycelia (SM) and conidia (SC) of *S. bambusicola* were collected as the control group and the experimental group, respectively. The BZ16 strain was inoculated on PDA medium and cultured for14 days. Refer to Zhang et al. (2016) method for counting conidia. When using solid culture, we injected 0.02% Tween-80 solution into the plate to be tested, which was shaken thoroughly. The solution containing conidia was obtained, which was centrifugated at 1,000 r/min for 15 min, and then counted under the light microscope with hemocytometer to calculate the average amount of conidia contained in each petri dish.

### Morphological observation and determination

The test tube slant of the strain was activated 2–3 times, seeded on Czapek medium and PDA medium, respectively, and cultured at 28°C. The culture characteristics were described and recorded daily. The mycelia were stained with lactophenol cotton blue (LPCB), and the number of conidia was counted using a hemocytometer under a light microscope. The conidia and mycelia of *S. bambusicola* were observed using the scanning electron microscopy (SEM).

### Protein extraction and preparation

We added sodium dodecyl sulfate (SDS) lysis solution to the sample, which was heated and ultrasonicated at high temperature to denature the protein. After the supernatant was centrifuged, we used trichloroacetic acid to precipitate the protein, which was washed with acetone and dried. Subsequently, we added 8 M urea/100 mM Tris–HCl solution (pH 8.0) to the protein precipitate to fully dissolve it. The supernatant was obtained after centrifugation at high speed for 15 min. Dithiothreitol was added to a final concentration of 10 mM, and the mixture was incubated at 37°C for 1 h. The sample was added with iodoacetamide to a final concentration of 40 mM and incubated at room temperature and in the dark. After adding an appropriate volume of 100 mM Tris–HCl solution (pH 8.0) to dilute the urea concentration to below 2 M, we added 1 μg of trypsin, and the mixture was incubated at 37°C with shaking for 12 h. Trifluoroacetic acid was added to terminate enzyme digestion, and the pH of the solution was adjusted to pH 6.0. After centrifugation at 12,000 × g for 15 min, we used a C18 column to desalt the peptides. After the peptide solution was drained by a centrifugal concentrator, it was frozen and stored at −20°C until it was tested on the machine.

### Mass spectrometry detection and database search

MS analysis was performed using the SCIEX TripleTOF 5600+ LC/MS system. Samples were separated by a liquid-phase Eksigent microLC 415 system at microliter flow rates. An analytical gradient was established using two mobile phases (mobile phase A: 3% DMSO, 0.1% formic acid, 97% H_2_O and mobile phase B: 3% DMSO, 0.1% formic acid, 97% ACN). The flow rate of the liquid phase was set to 5 μL/min. Peptide ions with signals exceeding 120 cycles per second initiated MS/MS scans.

MS data generated by TripleTOF 5600+ were retrieved using ProteinPilot. The database used for the search was a self-built proteome reference database. The search results were screened for qualified protein information based on Unused ≥ 1.3, and the remaining identification information was used for subsequent analysis. The differential proteome of conidiation formation from *S. bambusicola* strain BZ16 was obtained (*p* ≤ 0.05 and SC/SM fold change (FC) ≥ 1.5 or ≤0.067). Differential proteins were subjected to Gene Ontology (GO) functional annotation and Kyoto Encyclopedia of Genes and Genomes (KEGG) pathway analysis (Zhou et al., 2017; Niu et al., 2022; Choubey et al., 2023).

### Ethanol quantification assay

Protein was extracted from the two above-mentioned mycelial and conidial forms of *S. bambusicola* in standardized dry weights. The ethanol concentration in the extract was determined using an enzyme detection kit according to the manufacturer’s instructions (UV-test for ethanol, Shanghai Enzyme-linked Biotechnology Co., Ltd., China). Each sample had three replicates.

### Quantitative PCR analysis

Experiments were conducted from 0.20 g of the same weight of *S. bambusicola* in the above two forms. Each sample had three replicates. The forward and reverse primer sequences of differentially expressed genes were designed using the IDT primer design online tool (Supplementary Table 1). First, total RNA was extracted and reverse transcribed into cDNA, and then specific primers and SYBR Green I were used for quantitative PCR detection. The 18S gene was used as the internal reference gene and 2^−△△*Ct*^ was used to calculate the relative expression level. Glycoside hydrolase (*GH*) gene was selected to measure and analyze the expression differences related to the conidiospore formation of *S. bambusicola* (Kim et al., 2020).

### Data analysis

The experimental data were analyzed by ANOVA and Pearson’s correlation using SPSS software. Data results are expressed as mean ± standard deviation. Each experiment was repeated three times, and the statistical significance level was set at *p* < 0.05.

## Results

### Detection of conidiospore formation by *Shiraia bambusicola*

**Figure 1:**
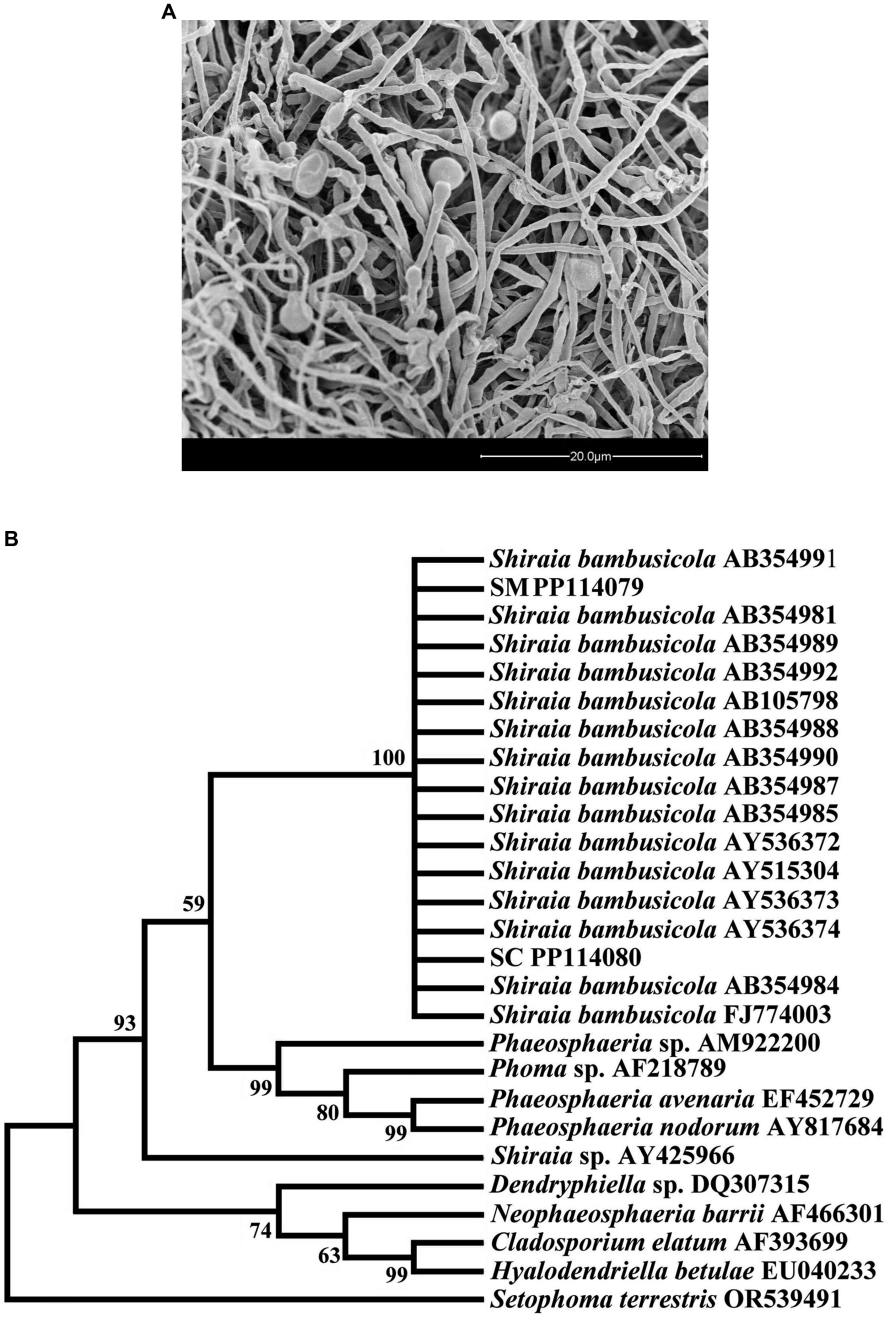
Observation and detection of conidia in *Shiraia bambusicola* **(A)** Observation of SC and SM; **(B)** Phylogenetic tree constructed by ITS of SC and SM.

Colony morphology observations revealed the growth of *S. bambusicola* in Czapek medium after 7 days. The aerial mycelium colonies were fluffy, and the back of the colonies was light to dark brown; after 14 days, the aerial mycelium became thinner and changed from white to light gray, and no conidia were observed. After culturing in PDA medium for 3 days, white colonies formed on the Petri dishes. After 7 days, the colonies expanded and became fluffy. The back of substrate mycelia colonies was dark brown, and green conidia were observed. After 14 days, the aerial mycelia became thinner, the color changed from white turned to light gray, and the volume of green conidia increased to (15.57 ± 0.71) × 10^6^/dish. Compared with Czapek medium, *S. bambusicola* grew faster and produced more conidia faster in PDA medium, with no obvious difference in colony characteristics. SEM revealed that the conidia were germinated, solitary, nearly round, and light in color (Figure 1A). Mucilage was observed on the surface of the conidia. After conidial production, the yield of hypocrellins decreased slightly, with no significant change.

The conidia produced by the culture of *S. bambusicola* were obtained, and the DNA was extracted to amplify the ITS sequence. Analytical observations via the neighbor-joining (NJ) method to establish a phylogenetic tree using Mega 7.0 software showed that the two samples of mycelia and conidia obtained through culture could be clustered with *S. bambusicola* downloaded from GenBank in a clade (Figure 1B) and showed 99% support. Molecular identification strongly demonstrated that the green oval conidia we observed were conidia of *S. bambusicola*.

### Basic identification of proteome

During the process of conidiospore formation from *S. bambusicola*, the SM and SC total proteins were pre-treated and subjected to specific restriction enzyme digestion to prepare a peptide solution, which was then desalted, enriched, and eluted by LC/MS from SCIEX. The collected SM and SC data were analyzed by ProteinPilot, and 48,747 secondary spectra within the global FDR 1% range were obtained, with 9,578 matching peptides. A total of 2,840 trusted proteins with unused≥1.3 were obtained from the SM and SC.

**Figure 2:**
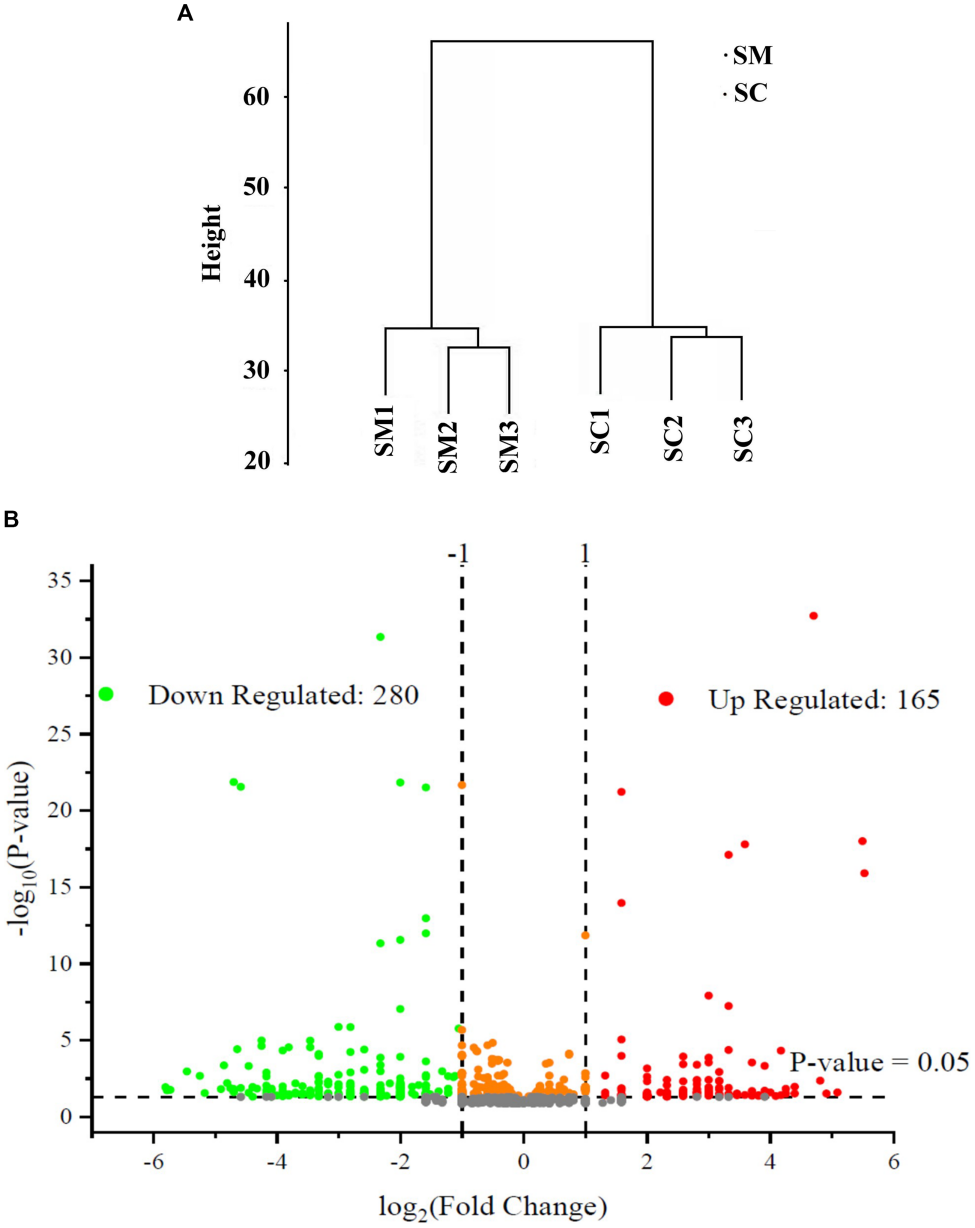
Quantitative quality control of the proteome and differential proteome analysis. **(A)** SC and SM are clustered into two different groups; **(B)** Distribution results of differentially expressed proteins (DEPs) between the SC and SM (Red are upregulated proteins, whereas green are downregulated proteins).

In this study, the expressed proteins in the conidiation of *S. bambusicola* strain BZ16 were quantified, and mycelia and conidia samples were collected. Three biological replicate samples were present in each group, with a total of six sets of data. Finally, 1,979 proteins were effectively quantified. After normalization of the protein peak areas of the SM group and SC group, the biological replicate samples of the SM group and SC group were clustered and belonged to two different groups (Figure 2A). The fold change (FC) distribution of the differentially expressed proteins (DEPs) is shown in a volcano plot (Figure 2B). After the t-test, 445 differentially expressed proteins were obtained between the SM group and SC group (*p* < 0.05 and SM/SC FC > 2.0), including 165 upregulated proteins and 280 downregulated proteins.

### GO functional annotation and enrichment

**Figure 3:**
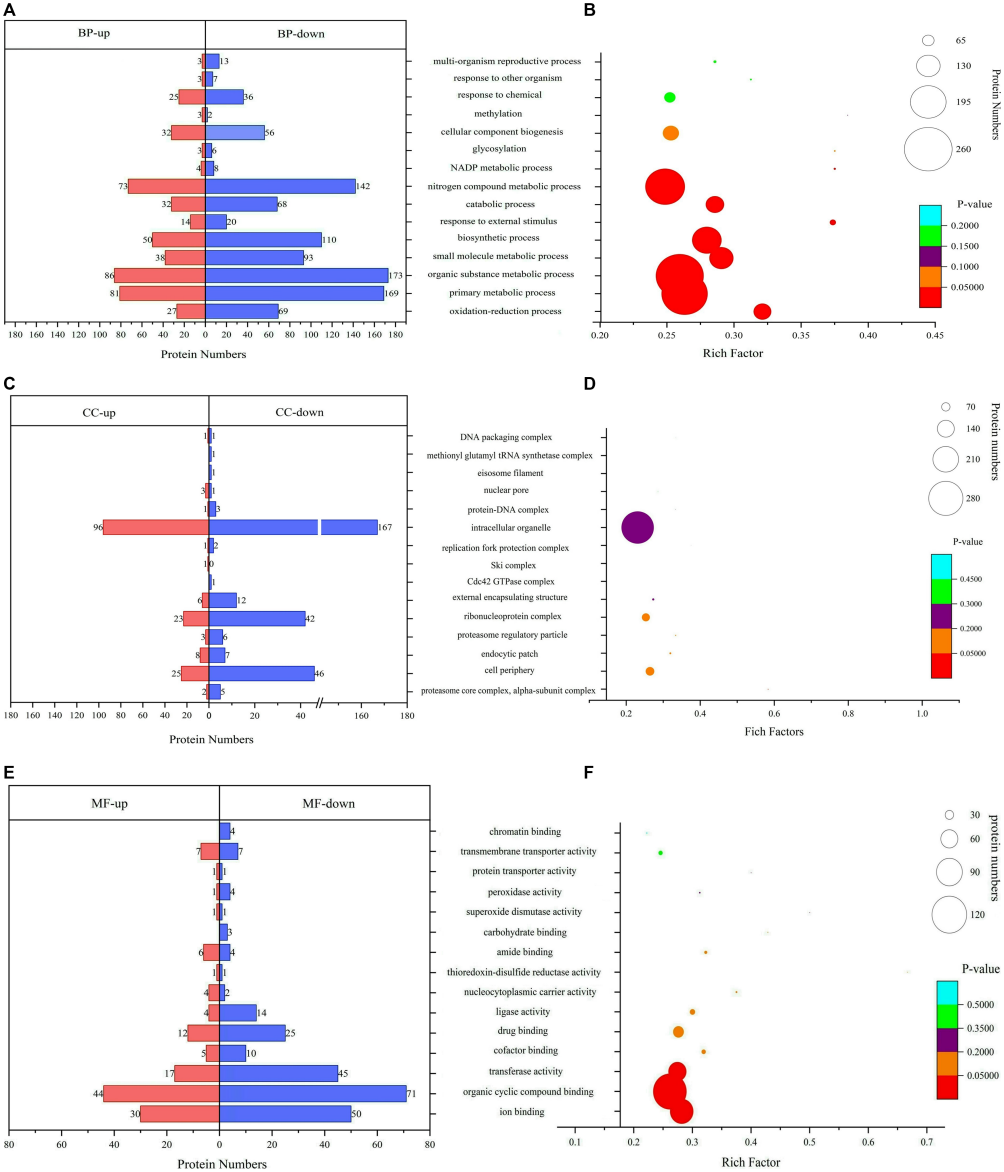
Gene Ontology (GO) functional classification and enrichment analyses. **(A)** Biological process (BP) annotation of DEPs. **(B)** Significantly enriched terms in BP. **(C)** Cellular component (CC) annotation of DEPs. **(D)** Significantly enriched terms in CC. **(E)** Molecular function (MF) annotation of DEPs. **(F)** Significantly enriched terms in MF.

The GO analysis and enrichment results of DEPs during the conidiospore formation from *S. bambusicola* strain BZ16 are shown in Figure 3. In the comprehensive analysis of protein significance of biological process (BP), molecular function (MF), and cellular component (CC), the BP was the most dominant in the number and significance of pathways (Supplementary Figure 1). Among the DEPs involved in the BP during conidiation (Figure 3A), the DEPs involved in the organic substance metabolic process accounted for 13.21, and 12.75% of DEPs were related to the primary metabolic process. The DEPs in the oxidation–reduction process were the most significantly enriched.

The CC of DEPs during the conidiation of *S. bambusicola* is diverse (Figure 3B). The most notable enrichment was observed in the proteasome core complex, specifically in the alpha-subunit complex, with *p* = 0.00766. This complex primarily contains various proteasome subunit alpha types, which participate in the metabolism of nitrogen compounds. The DEPs are also mainly distributed in the cell periphery and endocytic patch, which are used to form unique structures of conidia or reduce specific proteins that compose mycelia. The total number of DEPs in the cell periphery accounts for 12.16%, which mainly involves calcium regulatory proteins, transmembrane transport, protein localization and folding, signaling protein elongation, transport factors, molecular chaperones, and carbohydrate metabolism process. Among them, 11 DEPs are involved in polysaccharide metabolism, represented by UTP-glucose-1-phosphate uridylyltransferase (UGPases), 6-phosphogluconate dehydrogenase (6PGDH), chitin synthase (CHS1), GH, and other proteins, which are responsible for an increase in the biotransformation of cellulose, xylan, pectin, and arabinoxylan (Xiao et al., 2020).

The analysis results of DEPs involved in MF showed (Figure 3C) that 28.47% of DEPs participated in organic cyclic compound binding. The DEPs involved in ion binding accounted for 19.80%, the transferase activity accounted for 15.35%, and the enrichment value of ion binding was the most significant (Figure 3C). For example, calcium ion binding, calponin homology domain-containing protein, and GTPase increased significantly. GTPase is a class of GTP-binding proteins that play a molecular switching role in a variety of cellular reactions (Mann et al., 2016).

### KEGG pathway enrichment analysis

**Figure 4:**
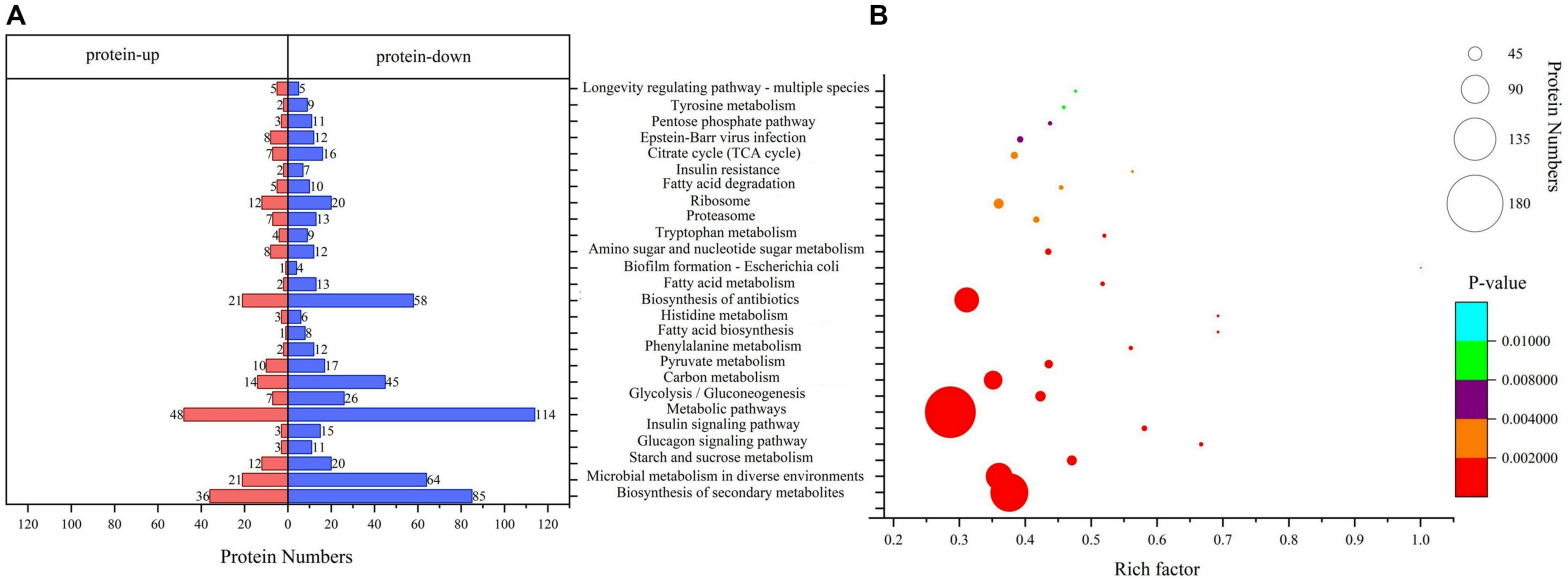
Kyoto Encyclopedia of Genes and Genomes (KEGG) pathway analysis. **(A)** KEGG pathway annotation of DEPs. **(B)** Significantly enriched functional annotations in KEGG pathways.

Pathway analysis is a direct method used to understand the biological processes and traits of cells systematically and comprehensively. The annotation and enrichment analysis results of the KEGG pathways showed (Figure 4) that a large number of proteins involved in the signal transduction pathways were expressed during conidiospore formation. Most of the differential proteins were found to participate in the metabolic process, particularly in the expression of genes related to carbon metabolism. The final growth and development changes focused on the various nutrients, hormone signals, and other essential substances for conidiospore formation.

Among them, the number of DEPs involved in the metabolic pathways was the largest. The enrichment of differential proteomes in the biosynthesis of secondary metabolites and microbial metabolism in diverse environments involves a broad spectrum of metabolic sites in protein distribution (Supplementary Table 2). Many pathways are related to saccharide metabolism, among which starch and sucrose metabolism have the most significant enrichment (*p* = 0.00000477), with 32 differential proteins involved in this process. Other pathways include glycolysis/gluconeogenesis, carbon metabolism, amino sugar and nucleotide sugar metabolism, and pentose phosphate pathway. Some pathways are related to the regulation of saccharide metabolism, such as the glucagon signaling pathway. This result is similar to the results we analyzed using the Clusters of Orthologous Groups (COG) database. The proteins in the carbohydrate transport and metabolism category have the most significant enrichment, with *p* = 0.000314 (Supplementary Figure 2).

### Proteins related to carbohydrate metabolism

**Figure 5:**
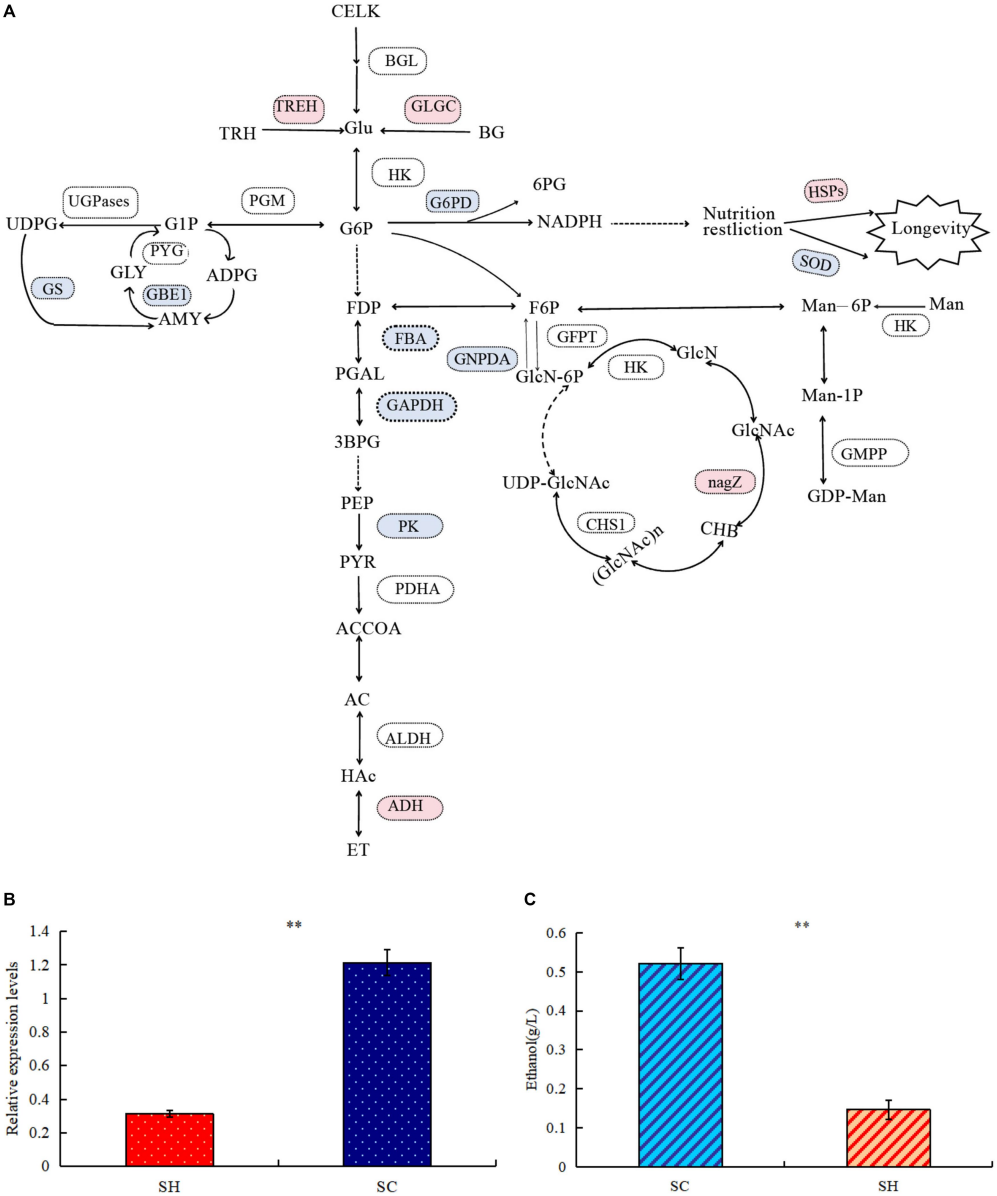
Comparison of protein profiles related to carbohydrates metabolisms in *Shiraia bambusicola* mycelia and conidia. **(A)** The diagram representing the carbohydrates metabolisms pathways represents differentially abundant proteins in the mycelia and conidia (Red are upregulated proteins, whereas blue are downregulated proteins). The list of significant expressed proteins are presented in Supplementary Tables 3, 4. **(B)** Quantitative PCR validations of *GH* genes. **(C)** Ethanol quantification assay. “**” indicates *p*-values < 0.01 by Student’s *t*-test.

Comparison of protein profiles related to carbohydrates metabolisms in SM and SC are shown in Figure 5A. Regarding the starch and sucrose metabolism pathways, the expression levels of glycogen synthase (GS) and 1,4-alpha-glucan-branching enzyme (GBE1) decreased in conidia, whereas the expression of GH and trehalase (TREH) increased. Thus, conidiospore formation may cause the glycogen content to decrease, whereas the amount of D-glucose increased and the branching degree of glycogen decreased. In this pathway, more differential nodes corresponded to multiple proteins with the same function, some of which were upregulated and some were downregulated. Examples of these proteins were phosphotransferase (PST), glucan phosphorylase (PYG), UGPase, phosphoglucomutase (PGM), and beta-glucosidase (BGL). Among the three significantly changed PGM, the expression of one was significantly upregulated and two were significantly decreased, which may cause changes in the phosphorylation position of glucose during conidiospore formation. In five PYG, the expression of A0A163ELL1 was significantly upregulated, and four were significantly decreased due to the decrease in the glycogen content. One type (A0A178B6A9) significantly increased in UGPases, and two types decreased significantly, which caused a relatively large change in the degree of phosphorylation of the product. Significant changes were also observed in the amino sugar and nucleotide sugar metabolism pathways, such as hexosaminidase (HEXA), glucose-6-phosphate isomerase (GPI), D-fructose-6-phosphate amidotransferase (GFPT), and chitin synthase (CHS). Their changes cause changes in the structure and content of polysaccharide products. GH has glucan 1,3-beta-glucosidase functions, belongs to the glycosyl hydrolase 5 family, and it may be involved in the processes of conidiospore formation. GH is characterized by its glucan-1,3-beta-glucosidase function, which may be involved in conidiation (Medina-Redondo et al., 2008). The results verified by quantitative PCR technology showed that the expression level of *GH* gene increased significantly by 3.89 times during conidiation (Figure 5B). This is similar to the proteome test results, both of which showed a significant increase. The difference in specific results may be caused by different methods and detection indicators.

In the glycolysis/gluconeogenesis pathway, the expression of the key enzymes fructose-bisphosphate aldolase, class II, and glyceraldehyde 3-phosphate dehydrogenase significantly decreased, resulting in reduction in the activity of the entire glycolysis/gluconeogenesis pathway. Pyruvate generated by the glycolysis of glucose will further degrade by aerobic respiration and anaerobic respiration. The expression levels of many enzymes in the tricarboxylic acid cycle were significantly reduced; in the ethanol fermentation of anaerobic respiration, the expression levels of many enzymes significantly increased. Among the three pyruvate dehydrogenase E1 component alpha subunits (PDHAs), two significantly increased. Alcohol dehydrogenase 1 (ADH1) significantly increased, resulting in active ethanol fermentation pathways, indicating abundance comparison of glycolytic and fermentation-related enzymes during conidiospore formation. Anaerobic respiration increases significantly during sporulation and ethanol production. To verify whether the mycelium of conidiospore formation is suitable for fermentation, we compared the ethanol production capacity of two species of *S. bambusicola*. During the culture process, the ethanol concentration in the conidial extract was determined to be almost 3.54 times that of the mycelial extract (*p* < 0.01), thereby confirming that the ability of conidial cells to obtain energy through fermentation metabolism was higher than that of mycelia (Figure 5C). This result was consistent with the findings of proteome analysis.

In the pentose phosphate pathway (HMP), the expression of glucose-6-phosphate 1-dehydrogenase (G6PD) decreased, leading to an decrease in the conversion of 6-phosphogluconate to gluconic acid. During this process, the released electrons are transferred to NADP (coenzyme II). Under restricted culture conditions, DNA can significantly change the expression of the longevity regulating pathway. The levels of superoxide dismutase (SOD) and SOD [Cu-Zn], which are both antioxidant enzymes, significantly decreased. Among the proteins examined, significant increases were observed in heat shock protein (HSP), HSP70 multi-domain protein, and HSP78. The conidial structure demonstrated an elevated presence of HSPs, which have the function of protecting their own stability. The stress-resistant reproductive function of conidia possibly enhanced cellular stability.

## Discussion

The successful biosynthesis of *S. bambusicola* must be accompanied with the application of conidia. In the cultivation process, conidia play an important role. If no conidia are produced, then fruiting bodies cannot form. Cultures with conidial production also do not necessarily have fruiting body formation. The mechanism of the conidia of *S. bambusicola* during its growth and development remains unclear. Therefore, acquiring the conidia of *S. bambusicola* and conducting in-depth research on them are important. This study observed green nearly round conidia with mucilage on their surface. The morphological observations revealed that the conidia of *S. bambusicola* were differentiated by the “blown” part from the top of the conidiophore. Other researchers also reported similar findings. Zhang et al. (2002) observed green spores, and Cai et al. (2008) used SEM to observe conidia similar to those in this experiment when performing liquid fermentation of *S. bambusicola*. We also performed the molecular identification of ITS. *Shiraia bambusicola* typically produces conidia during stress, forming structures that can withstand adverse environments and facilitate spread. Significant changes occur in the expression of many proteins, some of which are involved in forming conidial structures.

This study introduced quantitative proteomics to determine the proteome of the mycelia and conidia of *S. bambusicola*, and MS was conducted to detect the protein composition in the sample. The DEPs of mycelium components and spores were obtained by comparing the protein differences with the spectrogram counting method. Thus, GO annotation, KEGG metabolic pathway analysis, and protein–protein interaction analysis of DEPs were carried out, which revealed that the process of conidiospore formation from *S. bambusicola* was mainly due to carbohydrate metabolism. An increase in the synthesis and release of proteins related to ethanol and carbohydrate metabolism was conducive to the infection and spread of conidia of *S. bambusicola*.

GO annotation analysis showed significant changes in cell periphery, primary metabolic process, endocytic patch, and transferase activity. In the primary metabolic process, 250 DEPs were involved, of which 169 were reduced, accounting for 67.60%. In the conidiation of *S. bambusicola*, 280 proteins with down-regulated expression accounted for 62.93% among the DEPs. Conidiospore formation will cause a decrease in the total protein synthesis, which may be because the mycelia begins to enter the reproductive state to cope with the change in growth environment, the primary metabolism decreases, growth slows down, and biosynthesis begins to decline. In the cell periphery, 11 DEPs are involved in polysaccharide metabolism, which may cause an increase in biotransformation of substances such as cellulose, xylan, pectin, and arabinoxylan, and they may be associated with the perispore mucilage observed (Fu et al., 2019; Xiao et al., 2020). The main increased proteins were ADH1 and HSP70 multi-domain protein. The metabolic activity of alcohol dehydrogenase directly affects the metabolism of alcohol and the physiological state of the organism (Dash et al., 2019). Studies have found that the *MaADH1* gene in *Metarhizium anisopliae* promotes mycelial growth and sporulation through acetaldehyde detoxification under low oxygen conditions (Zhang et al., 2018). In this study, high levels of ethanol were detected during the growth stage of conidia, indicating increased oxygen-free respiration during conidial production, providing an intermediate for biosynthesis.

The conidia of *Aspergillus fumigatus* also had a significant increase in ADH protein content. ADH1 catalyzes the equilibrium redox conversion between acetaldehyde and ethanol, with the oxidation of acetaldehyde to ethanol being the energetically preferred reaction (Teutschbein et al., 2010). HSP70 multi-domain protein levels were very low in the mycelia of *S. bambusicola* but significantly increased during conidial formation. As a non-specific protective protein, HSP70 can help denatured proteins restore their correct conformation, facilitate the folding and assembly process of nascent peptide chains, and significantly improve the resistance of cells to stress (Kao et al., 2020). The differential proteins were also concentrated in the endocytic patch. The proteins whose expression levels increased significantly included calcium ion binding, Ca^2+^-binding actin-bundling protein, and calponin homology domain-containing proteins. They are widely distributed in cells as regulatory proteins, playing an important role in the calcium signaling pathway through reversible binding with calcium ions, regulating physiological metabolism and gene expression, and controlling normal cell growth and development (Ma et al., 2021). Endocytosis plays an important role in nutrient absorption and signal transduction of cells. The endocytotic protein EDE1, which is an early member of clathrin-mediated endocytosis in eukaryotes, is upregulated (Kozak and Kaksonen, 2022). The expression of beta-tubulin and tubulin alpha chain in the cytoskeleton is significantly reduced in intracellular organelles. It may represent a reduction in the cellular component required for sporulation by the biogenic component of *S. bambusicola*.

In the KEGG pathway analysis, the proteins in the carbohydrate transport and metabolism class demonstrated the most significant differences. Fifteen proteins comprise the GH class in the proteome sequence of *S. bambusicola*. Among them, GH and glycoside hydrolase family 1 (GH1) are the proteins that significantly increased. The monosaccharide composition of the sugar chain in the glycoside, the glycosidic bond configuration, and the glycoside connection method all affect the activity and metabolic pathways of the glycoside (Prieto et al., 2021; Drey et al., 2022). A significant increase in two cell wall glycosyl hydrolases, which function to remodel the cell wall between adjacent conidia to facilitate conidial formation and dissemination, was found in the conidia cell wall of *Neurospora crassa* (Pan et al., 2021). Glycoside hydrolase family 76 (GH76) protein is highly expressed in conidia, and is an important determinant of fungal growth, development and virulence in *Pyricularia oryzae* (Ao et al., 2016). However, no difference in GH was found in the conidia of many species of filamentous fungi (Xu et al., 2022; Cao et al., 2023).

Significant changes were also reported in the expression levels of trehalase and phosphotransferase in the conidia of *S. bambusicola*. Similar to previous reports, *Streptococcus pyogenes* can preferentially regulate carbohydrate utilization (such as glucose uptake), adapt to environmental changes, and change the expression of its own virulence factors by regulating the gene expression of the phosphoenol pyruvate-dependent phosphotransferase pathway (PTS) system (Valdes et al., 2018). GBE widely exists in prokaryotes and eukaryotes and is a key enzyme in catalyzing and regulating α(1–6) transglycosylation activity (Conchou et al., 2022). Significant changes were observed in the expression level of GBE1 in *S. bambusicola*. Glycogen branches can increase the water solubility of glycogen to facilitate glycogen storage and raise the number of non-reducing ends, thereby allowing biological organisms to aggregate when they need glycogen for energy. GBE regulates glycogen metabolism and participates in the response to abiotic stress (Yang et al., 2022b).

In the amino sugar and nucleotide sugar metabolism pathway, hexokinase expression changes significantly, resulting in fluctuations in the contents of mannose-6-phosphate (M6P), fructose-6-phosphate (F6P), and Glucose-6-phosphate (G6P). This phenomenon may be due to the presence of related monosaccharides in the spores structure. The synthesis of chitin in fungi is mainly concentrated in vigorous growth parts such as budding parts and hyphal tips (Takeshita, 2020). In *Saccharomyces cerevisiae*, chitin synthase plays an important role in cell damage repair, primary septum formation, septum ring formation, and cell wall formation (Zhao et al., 2019). In *Aspergillus nidulans*, part of chitin synthetase plays an important role in hyphal growth and septation production (Taheri-Talesh et al., 2012). We found that chitin synthase contains three significant proteases with the same function, two of which (A0A178AFR8) have increased content, which may be caused by the spherical structure of the conidia. The expression of many proteins fluctuates during the conidiospore formation of *S. bambusicola*. The increase of some polyglycosidases and the active metabolism of polysaccharides may be related to the differences in the composition of the unique structures of conidia, such as septa and extracellular mucilage. Mucilage from the conidia of *Hirsutella satumaensis* was also found to be related to chitin content, but there are still many proteins to be studied (Duan et al., 2021).

## Conclusion

In this study, the biological process of conidiation by *S. bambusicola* BZ16 was determined through various physical and chemical analyses, including morphological observations and molecular sequence detection. The proteomics response was detected during the conidiospore formation of *S. bambusicola*, which mainly revealed the CC changes in proteasome core complex, cell periphery, endocytic patch, and so on. The proteins with the most significant differences were mainly concentrated in the carbohydrate transport and metabolism pathway. The proteins involved included ADH1, GH, BGL, HSP70 multi-domain protein, ubiquitin, malate synthase, thioredoxin, elongation factor Tu, alpha-1,2-mannosidase, and so on. The content of GH and ethanol were also detected, and it was found that conidiospore formation could promote their production. Future research needs to further elucidate these mechanisms, provide a basis for the conidiation mechanism, lay a theoretical foundation for the industrial utilization of *S. bambusicola*, and explore the sexual reproduction of fungi.

## Data availability statement

The datasets presented in this study can be found in online repositories. The names of the repository/repositories and accession number(s) can be found in the article/Supplementary material.

## Author contributions

WD: Conceptualization, Data curation, Formal analysis, Funding acquisition, Methodology, Project administration, Resources, Software, Supervision, Validation, Writing – original draft, Writing – review & editing. CS: Conceptualization, Data curation, Formal analysis, Funding acquisition, Methodology, Project administration, Resources, Software, Supervision, Validation, Writing – review & editing. TW: Conceptualization, Formal analysis, Writing – review & editing. WL: Conceptualization, Data curation, Writing – review & editing. BD: Conceptualization, Writing – review & editing. BW: Conceptualization, Funding acquisition, Writing – review & editing. SS: Software, Writing – review & editing. QY: Software, Writing – review & editing. WH: Project administration, Writing – review & editing. SC: Project administration, Writing – review & editing.
