# Supplementary material for: Comparative proteomics analysis of Shiraia bambusicola revealed a variety of regulatory systems on conidiospore formation
Source: Front Microbiol. 2024 May 22;15:1373597. doi: 10.3389/fmicb.2024.1373597 (PMC11152172; doi:10.3389/fmicb.2024.1373597)
Supplement: Supplementary file 1 [file Data_Sheet_1.PDF]

Supplementary Table 1 Primers for the gene transcription level analysis. F: forward primer, R: reverse primer.

Genes	Gene description	Name of primer	Sequence
18S	Reference gene	18S-F	5'-ACGCAGCGAAATGCGATAAG-3'
		18S-R	5'-CAAATTGTGCTGCGCTCCAA-3'
GH	Glycosyl hydrolase	GH-F	5'-CGATAACTCTGGCAAGTACGG-3'
		GH-R	5'-AAGGACTTTGATGGTGTAGGC-3'

Supplementary Figure 1. Enrichment analysis of proteins with significant differences in biological process (BP), Molecular Function (MF), and Cellular Component (CC)

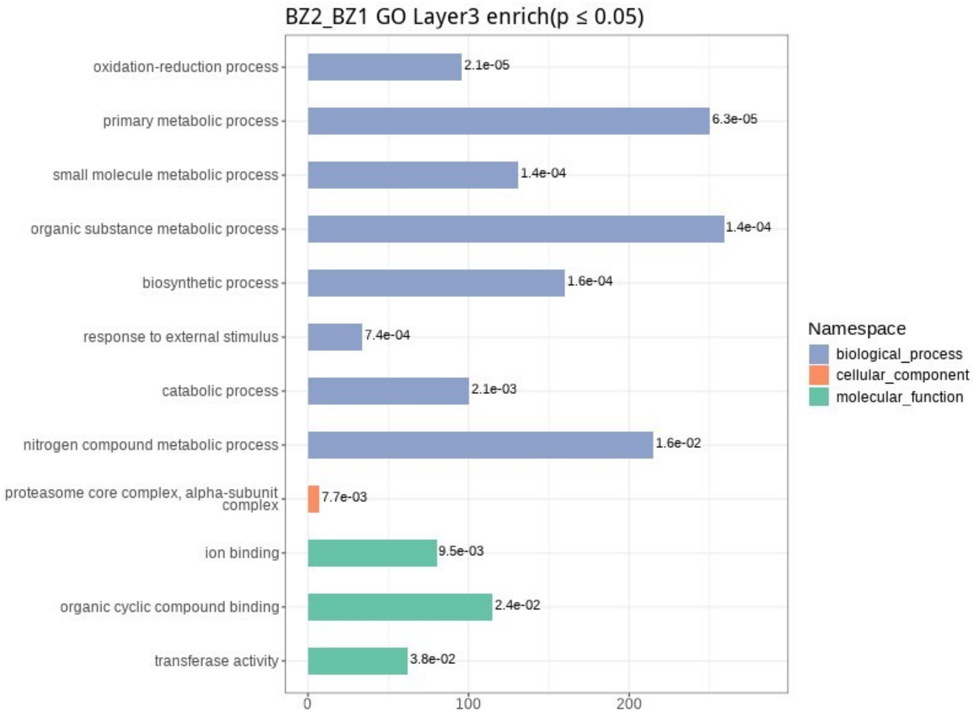

Supplementary Table 2 The number and type of proteins included in Biosynthesis of secondary metabolites and Microbial metabolism in diverse environments.

MapPathway	Description	QeRatio	BgRatio	Accession-down	Accession-up
map01110	Biosynthesis of secondary metabolites	121|445	322|1976	A0A163IRJ0;A0A163JF20;A0A177C705;A0A177CFI0;A0A177DCP0;A0A177DDX2;A0A177DS52;A0A177DVU7;A0A178AIV7;A0A178ARW4;A0A178AS63;A0A178AST7;A0A178ATV8;A0A178AY20;A0A178AYB5;A0A178BAE5;A0A178BCI8;A0A178DQS0;A0A178DVV6;A0A178DX47;A0A178DXJ7;A0A178E0H4;A0A178EFB6;A0A1Y2LKQ9;A0A1Y2MFP4;A0A2T2NCH7;A0A2T2P7V1;A0A2V1DJQ5;A0A2V1DL51;A0A2W1EVD9;A0A2W1HPY9;A0A2W1HQN7;A0A364MZI6;A0A386YT08;A0A399HRK9;A0A6A5K087;A0A6A5K543;A0A6A5K5C0;A0A6A5KGK5;A0A6A5KTY2;A0A6A5Q6N0;A0A6A5QK02;A0A6A5QRV1;A0A6A5QUM0;A0A6A5R2F2;A0A6A5RHK8;A0A6A5RHX9;A0A6A5RV32;A0A6A5SSL5;A0A6A5T1Q9;A0A6A5VAE7;A0A6A5XMK9;A0A6A5Y4P6;A0A6A5Z9T5;A0A6A6A9P5;A0A6A6ACE9;A0A6A6AF55;A0A6A6IXG6;A0A6A6J5I2;A0A6A6JZ70;A0A6A6SM26;A0A6A6VIB5;A0A6A6XAM9;A0A6A6XWT2;A0A6A6ZDW5;A0A6A6ZJH5;A0A6A6ZVI2;A0A6A6ZZX6;A0A6A7A9Y2;A0A6A7ACD5;A0A6A7B9I9;A0A6A7BLN2;A0A6G1K9F5;A0A6G1KDF4;B6CUW8;E4ZGY0;E5A0C4;E5A0D7;M2SNZ5;M2T526;M2TBY3;Q0UMT9;Q0UXB3;Q0V2F1;W6YN14	A0A163ELL1;A0A163GH77;A0A177CDZ1;A0A177CU03;A0A178AE01;A0A178BCU4;A0A178DPA4;A0A178EGQ7;A0A1Y2LX81;A0A2V1DUM0;A0A2W1CQ74;A0A364N088;A0A4V1X0G7;A0A6A5KLD2;A0A6A5Q4V6;A0A6A5S1H2;A0A6A5SKH4;A0A6A5U900;A0A6A5Z7T9;A0A6A5ZTM8;A0A6A6JNW0;A0A6A6JPG8;A0A6A6JSS8;A0A6A6TEL1;A0A6A6WQX0;A0A6A6XJ68;A0A6A7A6T7;A0A6A7BDW9;A0A6A7BMD3;A0A6G1JK69;A0A6G1K1G5;B6DQL3;E4ZLH0;M2TAU2;Q0UIH7;R0KM51

map01120	Microbial metabolism in diverse environments	85|445	236|1976	A0A163IQW3;A0A163IRJ0;A0A163IST0;A0A177C705;A0A177CFI0;A0A177DS52;A0A178ARW4;A0A178AS63;A0A178AST7;A0A178ATV8;A0A178BAE5;A0A178BCI8;A0A178DQS0;A0A178DVV6;A0A178DX47;A0A178E0H4;A0A178EFB6;A0A178EMT5;A0A1Y2LKQ9;A0A1Y2MDB8;A0A1Y2MFP4;A0A2T2N4K9;A0A2T2NCH7;A0A2T2P7V1;A0A2V1DJQ5;A0A2V1DL51;A0A2W1DNH3;A0A2W1EVD9;A0A2W1HQN7;A0A364MZI6;A0A386YQQ1;A0A386YT08;A0A399HRK9;A0A6A5K087;A0A6A5K543;A0A6A5K5C0;A0A6A5KGK5;A0A6A5KTY2;A0A6A5Q6N0;A0A6A5QRV1;A0A6A5QUM0;A0A6A5R2F2;A0A6A5RAD9;A0A6A5RV32;A0A6A5VAE7;A0A6A5XMK9;A0A6A6A9P5;A0A6A6ACE9;A0A6A6IXG6;A0A6A6J5I2;A0A6A6S771;A0A6A6ZVI2;A0A6A6ZZX6;A0A6A7A9Y2;A0A6A7BLN2;B6CUW8;E4ZGY0;E5A0C4;E5A0D7;M2TBY3;Q0UMT9;Q0V2F1;Q0V7E2;W6YN14	A0A162ZFW0;A0A177CDZ1;A0A177CU03;A0A178DPA4;A0A178EGQ7;A0A2W1DVA5;A0A364N088;A0A3M7MIH7;A0A4V1X0G7;A0A6A5S1H2;A0A6A5ZTM8;A0A6A6JNW0;A0A6A6JPG8;A0A6A6TEL1;A0A6A6WQX0;A0A6G1K1G5;B6DQL3;M2TAU2;Q0UIH7;Q0UYY5;R0KM51

Supplementary Figure 2 Enrichment of differential proteins analyzed using the COG database

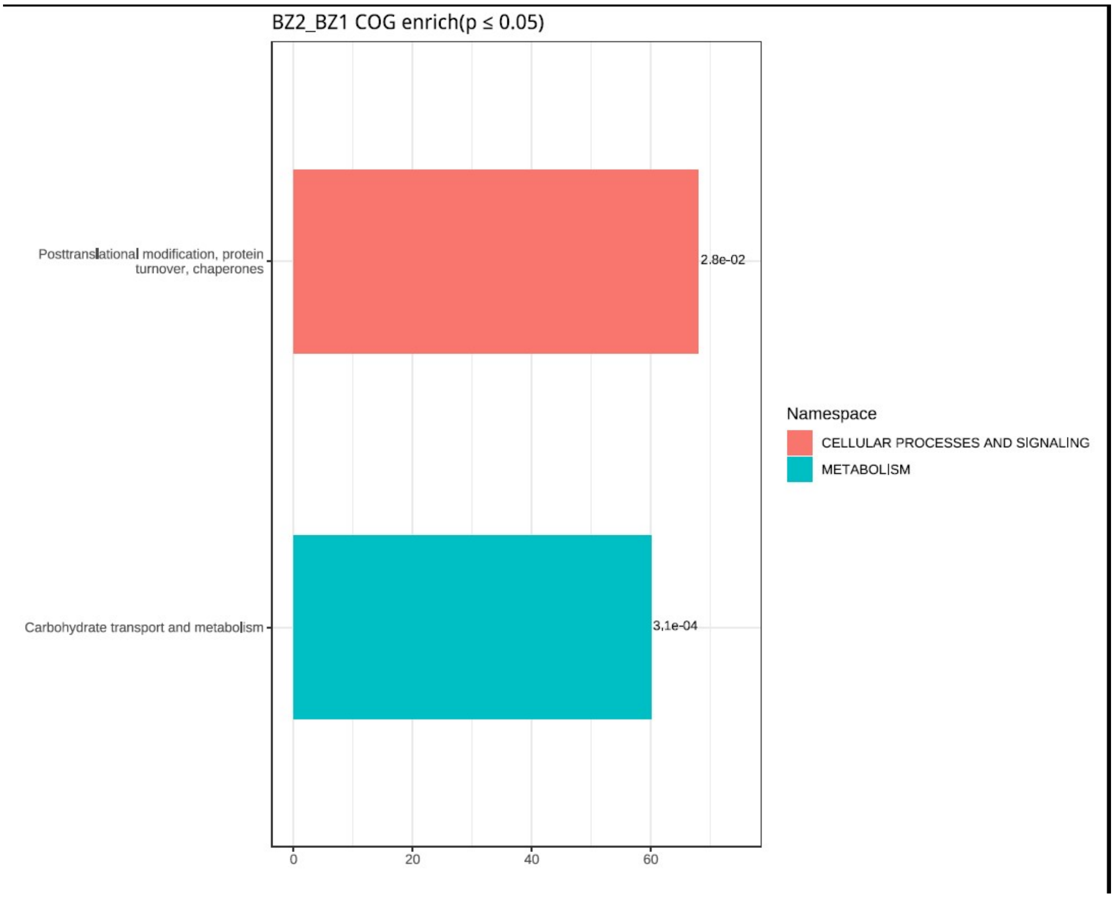

Supplementary Table 3 Significant expressed proteins related to carbohydrates metabolisms in *S. bambusicola* mycelium and conidia

Uniprot ID	Protein annotation	log2(SC/SM)	Abbreviation
A0A178A9E3	Glycoside hydrolase (MAP: glucan 1,3-beta-glucosidase [EC:3.2.1.58])	3.9069+	GH
A0A163D877	Trehalase(alpha,alpha-trehalase [EC:3.2.1.28])	2.3219+	TREH
A0A6A6RQT7	Trehalase (alpha,alpha-trehalase [EC:3.2.1.28])	3+	
E4ZWF6	Beta-hexosaminidase ([EC:3.2.1.52])	2+	HEXA
A0A177DYU1	Heat shock protein(clpB; ATP-dependent Clp protease ATP-binding subunit ClpB)	2.585++	HSP
A0A6A6HQL8	Heat shock protein 78 (ENTPD2; adenosinetriphosphatase [EC:3.6.1.3] | clpB; ATP-dependent Clp protease ATP-binding subunit ClpB)	2.3219++	
A0A4V1X0G7	Alcohol dehydrogenase 1(alcohol dehydrogenase, propanol-preferring [EC:1.1.1.1])	4.1699++	ADH1
A0A177CAF0	Glycogen [starch] synthase(glycogen synthase [EC:2.4.1.11])	1-	GS
E5A9B8	Glycogen [starch] synthase(glycogen synthase [EC:2.4.1.11])	3.4594--	
A0A2W1HPY9	1,4-alpha-glucan-branching enzyme([EC:2.4.1.18])	3.585--	GBE1
A0A6A6AF55	1,4-alpha-glucan-branching enzyme([EC:2.4.1.18])	2.3219--	
A0A178AS63	Fructose-bisphosphate aldolase (beta-mannosidase [EC:3.2.1.25]; fructose-bisphosphate aldolase, class II [EC:4.1.2.13])	1.1155-	FBA
A0A6A5K087	Fructose-bisphosphate aldolase (beta-mannosidase [EC:3.2.1.25]; fructose-bisphosphate aldolase, class II [EC:4.1.2.13])	2.3219--	
B6CUW8	Glyceraldehyde-3-phosphate dehydrogenase (Fragment) [EC:1.2.1.12]	1.1375-	GAPDH
E4ZGY0	Glyceraldehyde-3-phosphate dehydrogenase [EC:1.2.1.12]	0.9386-	

A0A6A6IXG6	Pyruvate kinase([EC:2.7.1.40])	2.3219--	PK
A0A6A6ZES8	Superoxide dismutase(SOD2; superoxide dismutase, Fe-Mn family [EC:1.15.1.1])	1.4594--	SOD
A0A178ASJ0	Glucosamine-6-phosphate isomerase ([EC:3.5.99.6])	3--	GNPDA
A0A178DX47	Glucose-6-phosphate isomerase ([EC:5.3.1.9])	4.3219--	GPI
A0A6A6A9P5	Glucose-6-phosphate 1-dehydrogenase([EC:1.1.1.49 1.1.1.363])	4.1699--	G6PD

Supplementary Table 4 The abbreviation for the name of compounds

Name of compounds	Abbreviation
Cellulose	CELK
Glucose	Glu
Trehalose	TRH
1,3-β-glucan	BG
Glucose-6-phosphate	G6P
Glucose-1-phosphate	G1P
UDP-glucose	UDPG
Amylose	AMY
Glycogen	GLY
ADP-glucose	ADPG
D-fructose1,6-diphosphate	FDP
gluconate 6-phosphate	Gluc6P
Mannose	Man
Mannose-6-phosphate	M6P
Mannose-1-phosphate	M1P
Fructose-6-phosphate	F6P
Glyceraldehyde 3-phosphate	PGAL
Glycerate-1,3-bisphosphate	BPGA
Phosphoenolpyruvate	PEP
Pyruvate	PYR
Acetyl-CoA	ACCOA
Acetate	ACE
Acetaldehyde	HAc
Ethanol	ET
Nicotinamide adenine dinucleotide phosphate hydride	NADPH

Guanosine diphosphate	GDP
Guanosine diphosphate mannose	GDP-Man
D-glucosamine-6-phosphate	GlcN-6P
UDP-N-acetylglucosamine	UDP-GlcNAc
Chitin	$(GlcNAc)_n$
Chitobiose	CHB
Acetyl D-glucosamine	GlcNAc
Beta-glucosidase	BGL
Glucose-6-phosphate-1-dehydrogenase	G6PD
Beta-glucosidase	BGL
Phosphotransferase	PST
Phosphoglucomutase	PGM
UTP--glucose-1-phosphate uridylyltransferase	UGPases
Alpha-1,4 glucan phosphorylase	PYG
Pyruvate dehydrogenase E1 component subunit alpha	PDHA
Aldehyde dehydrogenase	ALDH
Chitin synthase	CHS1
GDP-mannose pyrophosphorylase	GMPP
D-fructose-6-phosphate amidotransferase	GFPT
Glycoside hydrolase	GH
UTP-glucose-1-phosphate uridylyltransferase	UGPases
6-phosphogluconate dehydrogenase	6PGDH
chitin synthase	CHS

glycogen synthase	GS
Beta-hexosaminidase	HEXA
Glucose-6-phosphate isomerase	GPI
D-fructose-6-phosphate amidotransferase	GFPT
Glucose-6-phosphate 1-dehydrogenase	G6PD
Superoxide dismutase	SOD
Glucan-branching enzyme	GBE
